# Structure of GTP cyclohydrolase I from *Listeria monocytogenes*, a potential anti-infective drug target

**DOI:** 10.1107/S2053230X19010902

**Published:** 2019-08-30

**Authors:** Sonja Schüssler, Ilka Haase, Markus Perbandt, Boris Illarionov, Alexandra Siemens, Klaus Richter, Adelbert Bacher, Markus Fischer, Tobias Gräwert

**Affiliations:** a Hamburg School of Food Science, Universität Hamburg, Grindelallee 117, 20146 Hamburg, Germany; b Institute for Biochemistry and Molecular Biology, Laboratory for Structural Biology of Infection and Inflammation, Universität Hamburg, Notkestrasse 85, 22607 Hamburg, Germany; c The Hamburg Center for Ultrafast Imaging, Universität Hamburg, Luruper Chaussee 149, 22761 Hamburg, Germany; d Department of Chemistry, Technical University of Munich, Lichtenbergstrasse 4, 85748 Garching, Germany; e Hamburg Outstation, European Molecular Biology Laboratory Hamburg, Notkestrasse 85, 22607 Hamburg, Germany

**Keywords:** GTP cyclohydrolase I, *Listeria monocytogenes*, crystal structure, high-throughput screening, tetrahydrofolate biosynthesis, listeriosis

## Abstract

A putative open reading frame encoding GTP cyclohydrolase I from *Listeria monocytogenes* was expressed in a recombinant *Escherichia coli* strain. The recombinant protein was purified and was confirmed to convert GTP to dihydroneopterin triphosphate (*K*_m_ = 53 µ*M*; *v*_max_ = 180 nmol mg^−1^ min^−1^). The protein was crystallized from 1.3 *M* sodium citrate pH 7.3 and the crystal structure was solved at a resolution of 2.4 Å (*R*_free_ = 0.226) by molecular replacement using human GTP cyclohydrolase I as a template. The protein is a *D*_5_-symmetric decamer with ten topologically equivalent active sites. Screening a small library of about 9000 compounds afforded several inhibitors with IC_50_ values in the low-micromolar range. Several inhibitors had significant selectivity with regard to human GTP cyclohydrolase I. Hence, GTP cyclohydrolase I may be a potential target for novel drugs directed at microbial infections, including listeriosis, a rare disease with high mortality.

## Abstract 2

GTP cyclohydrolase I from *Listeria monocytogenes*, a putative anti-infective drug target, has been crystallized and the crystal structure was solved at 2.4 Å resolution.

## 1. Introduction   

GTP cyclohydrolase I (EC 3.5.4.16) catalyzes a mechanistically complex ring expansion whereby GTP is converted to dihydroneopterin triphosphate (CAS Registry No. 20574-65-6; Supplementary Fig. S1; Yim & Brown, 1976 ▸; Burg & Brown, 1968 ▸; Fukushima *et al.*, 1977 ▸; for a review, see Gräwert *et al.*, 2013 ▸). More specifically, the imidazole ring of the substrate is hydrolytically opened with the assistance of an essential zinc ion (Bracher *et al.*, 2001 ▸), and the resulting pyrimidine intermediate undergoes an Amadori rearrangement of the carbohydrate side chain followed by ring closure (Bracher *et al.*, 1998 ▸; Rebelo *et al.*, 2003 ▸; Tanaka *et al.*, 2005 ▸). The enzyme product, dihydroneopterin triphosphate, serves as the first committed intermediate in the biosynthesis of tetrahydro­folate by many microorganisms and as the first committed intermediate in the biosynthesis of tetrahydro­biopterin by animals (Gräwert *et al.*, 2013 ▸).

**Figure 1:**
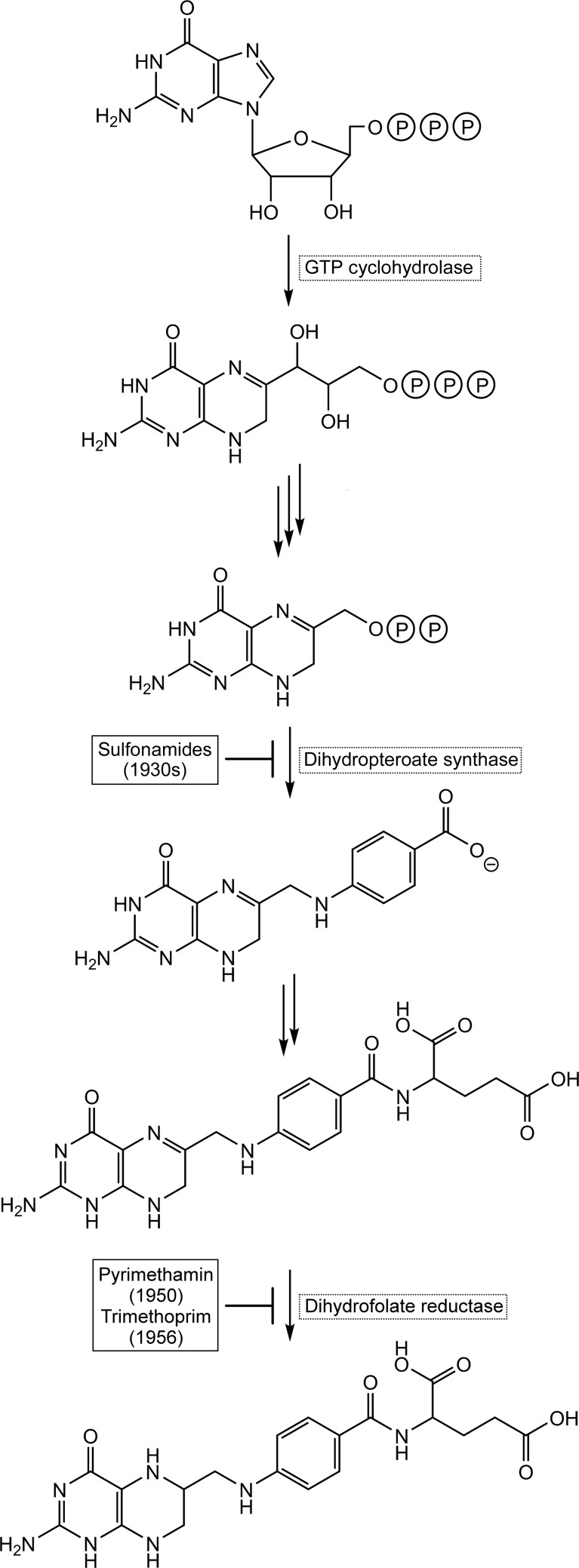
Bacterial tetrahydrofolate biosynthesis. Enzymes that are existing targets or are potential new targets are shown in black dotted rectangles. Antibiotics inhibiting the enzymes of this pathway are shown near their targets.

Inhibitors of tetrahydrofolate biosynthesis have played important roles in the treatment of a wide variety of microbial and protozoan infections (Fig. 1 ▸; Brown, 1962 ▸; for a review, see Swarbrick *et al.*, 2008 ▸). Specifically, sulfonamide drugs inhibiting dihydropteroate synthase, the penultimate enzyme of the dihydrofolate pathway, came to the market in the 1930s as the first synthetic agents with broad antimicrobial activity (Nobel Prize awarded to Gerhard Domagk in 1939; Nobel Media AB, 2014*a* ▸). Whereas sulfonamides have been superseded by more recently developed drugs in the treatment of bacterial infections, they continue to be relevant for certain protozoan diseases such as toxoplasmosis and drug-resistant malaria.

The subsequent development of pyrimethamin (in 1950) and trimethoprim (in 1956), which are inhibitors of dihydro­folate reductase, was also honored with Nobel Prizes to Gertrude Elion and George Hitchings in 1988 (Nobel Media AB, 2014 ▸ *b*); these authors also pioneered the combination of two antifolates directed at two different targets for improved antimicrobial activity.

During the ensuing heyday of the emerging antibiotics era, the emphasis in the field shifted away from synthetic compounds in the direction of natural and semisynthetic agents, while the elucidation of the tetrahydrofolate pathway in the second half of the 20th century has so far not been conducive to the discovery of novel antifolate drugs (for a review, see Burg & Brown, 1968 ▸).

This paper describes the structure elucidation of GTP cyclohydrolase I from *Listeria monocytogenes*, the foodborne causative agent of listeriosis, which has a mortality rate in the region of 50% (Lorber, 1997 ▸; Schlech & Acheson, 2000 ▸; Granier *et al.*, 2011 ▸). At present, listeriosis infections are usually treated with ampicillin. Cephalosporins are ineffective in *Listeria* and thus no substitute is available in the case of sensitivity towards β-lactam antibiotics or in the case of emerging resistance. A potential role of the enzyme as an anti-infective drug target is suggested by the discovery of inhibitors which might serve as lead structures, probably also against other pathogens.

## 2. Materials and methods   

### 2.1. Macromolecule production   

#### 2.1.1. Gene cloning and bacterial culture   

The putative *folE* gene from *L. monocytogenes* (ATCC BAA-679, LGC Standards GmbH, Wesel, Germany) was amplified by two consecutive PCR cycles using the primer pair Fw-LmGTPCHI and Bw-BamHI-LmGTPCHI and the primer pair Fw-EcoRI-LmGTPCHI and Bw-BamHI-LmGTPCHI. The amplificate was digested with EcoRI and BamHI and was ligated into the plasmid pNCO113 that had been treated with the same restriction enzymes. The resulting plasmid pNCO-His_6_-EK-LmGTP-CHI was transformed into chemically competent XL1-Blue cells (Bullock *et al.*, 1987 ▸; Stratagene, Amsterdam, The Netherlands), affording strain XL1 pNCO-His_6_-EK-LmGTPCHI. Strain XL1 pNCO-His_6_-EK-LmGTP-CHI was grown in Terrific Broth containing 170 mg ampicillin per litre for 4 h at 37°C and for a further 36 h at 30°C. The cells were harvested by centrifugation at 4°C and 4000 rev min^−1^ for 30 min, washed with saline [0.9%(*w*/*v*) NaCl] and stored at −20°C.

#### 2.1.2. Protein purification   

All steps were carried out at 4°C unless stated otherwise. The cells were thawed on ice, resuspended in buffer *A* (50 m*M* Tris–HCl pH 8.0 containing 250 m*M* NaCl) and disrupted by sonication. The mixture was centrifuged and the supernatant was loaded onto a nickel–NTA agarose column (30 ml; Macherey-Nagel, Düren, Germany) that had been equilibrated with buffer *A*. The column was washed with 300 ml buffer *A* followed by 100 and 500 m*M* imidazole in buffer *A*. Fractions were combined and concentrated by ultrafiltration. The solution was applied onto a Superdex 200 prep-grade column (5.3 cm^2^ × 60 cm), which was developed with buffer *A*. Fractions were combined and concentrated by ultrafiltration. Dithiothreitol was added to a final concentration of 2 m*M* and the protein was stored at −80°C. Human GTP cyclohydrolase I protein was prepared as reported previously (Auerbach *et al.*, 2000 ▸). Macromolecule-production information is summarized in Table 1 ▸.

### 2.2. Crystallization   

Crystals were grown by the sitting-drop vapor-diffusion method at 20°C. In initial screening, the Index, Crystal Screen and Crystal Screen 2 kits (Hampton Research, Aliso Viejo, California, USA) were used with a final protein concentration of 5 mg ml^−1^. The protein was supplied in 10 m*M* Tris–HCl pH 7.0 containing 75 m*M* NaCl. Aliquots (1 µl) of protein solution were mixed with 1 µl reservoir buffer solution to obtain a sitting drop. The mother-liquor reservoir contained 60 µl reservoir buffer solution. The protein crystallized from 1.33 *M* sodium citrate pH 7.3 containing 0.1 *M* HEPES. Ortho­rhombic crystals (space group *P*2_1_, unit-cell parameters *a* = 78, *b* = 142, *c* = 91 Å, 47% solvent content) grew to final dimensions of about 400 × 500 × 300 µm. Crystallization information is summarized in Table 2 ▸.

### 2.3. Data collection and processing   

Prior to data collection, crystals were soaked in glycerol for 10 min and were subsequently shock-frozen in a nitrogen stream at 100 K (Oxford Cryosystems). A native data set was collected on the P14 beamline at DESY, Hamburg, Germany. The *XDS* program package (Kabsch, 2010 ▸) was used to process the collected data. The crystals diffracted to a resolution of 2.4 Å. Data-collection and processing statistics are summarized in Table 3 ▸.

### 2.4. Structure solution and refinement   

Crystal structure analysis was performed by molecular replacement with *Phaser* (McCoy *et al.*, 2007 ▸) within the *CCP*4 package (Winn *et al.*, 2011 ▸) using the atomic coordinates of human GTP cyclohydrolase I (PDB entry 1fb1; Auerbach *et al.*, 2000 ▸) as a template. TLS refinement including non­crystallographic symmetry averaging was performed with the *PDB-REDO* server (Joosten *et al.*, 2014 ▸). The electron density and model were improved by successive rounds of model building and subsequent refinement using *Coot* (Emsley *et al.*, 2010 ▸) and *REFMAC*5 (Murshudov *et al.*, 2011 ▸). The asymmetric unit contains one decamer of GTP cyclohydrolase I. 15 N-terminal residues (MHHHHHHGSDDDDKE) and one C-terminal residue (N) are disordered and thus are missing from the model in every monomer. *MolProbity* (Chen *et al.*, 2010 ▸) was used for Ramachandran analysis. The atomic coordinates and structure factors of GTP cyclohydrolase I from *L. monocytogenes* have been deposited in the PDB as entry 4uqf. Refinement statistics are summarized in Table 4 ▸.

## 3. Results and discussion   

### 3.1. Results   

A putative *folE* gene encoding GTP cyclohydrolase I was amplified from *L. monocytogenes* DNA by PCR (Mullis *et al.*, 1986 ▸) and was cloned into the pNCO113 plasmid (Stüber *et al.*, 1990 ▸), where it was preceded by sequence elements encoding a polyhistidine tag and an enterokinase cleavage site. After transformation into a recombinant *Escherichia coli* host strain, the plasmid directed the synthesis of a polypeptide with an approximate mass of 22 kDa, as estimated by SDS–PAGE (Laemmli, 1970 ▸), which was in good agreement with the calculated mass of 22.9 kDa. The recombinant protein was isolated by metal-affinity chromatography. The predicted sequence was confirmed by partial Edman degradation (Edman, 1960 ▸) and by mass spectrometry of a tryptic digest (Mann & Wilm, 1994 ▸).

The recombinant protein catalyzes the conversion of GTP to dihydroneopterin triphosphate (*v*_max_ = 180 nmol mg^−1^ min^−1^, *K*_m_ = 53 µ*M*; Supplementary Fig. S3) as determined by photometric analysis (for details of the enzyme assay, see the supporting information). The formation of the second enzyme product, formate, was also directly detected by NMR spectroscopy using [U-^13^C_10_]GTP as substrate. In line with reports on other known GTP cyclohydrolase I orthologs, the addition of divalent cations to the assay mixtures was not required for activity.

Equilibrium sedimentation yielded a molecular weight of 212 ± 30 kDa. Boundary sedimentation resulted in a sedimentation velocity of 9.6 S. Mass spectrometry revealed the presence of 0.9 moles of zinc per mole of polypeptide.

**Figure 2:**
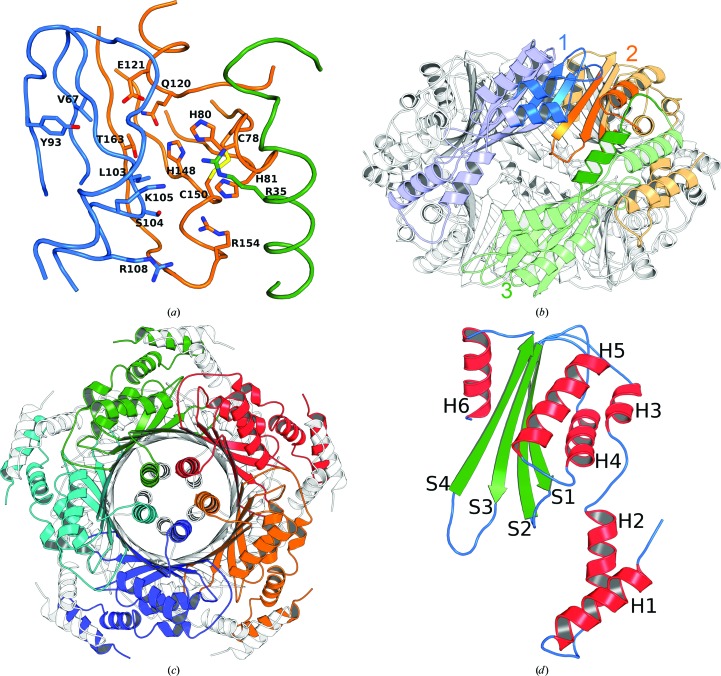
Structural features of *L. monocytogenes* GTP cyclohydrolase I. (*a*) Close-up of the active site. Conserved amino acids are shown in stick representation. (*b*) Active-site composition; the view is along one *C*_2_ axis. Monomer 1 (blue) and monomer 2 (orange) belong to the ‘top’ pentamer; monomer 3 (green) belongs to the ‘bottom’ pentamer. Intense coloring indicates the active site (one of ten) formed by these monomers. (*c*) View along the *C*_5_ axis. Monomers of the ‘top’ pentamer are shown in distinct colors and the ‘bottom’ pentamer is in black and white. (*d*) Monomer architecture. H, helix; S, strand. These figures were prepared using *PyMOL* (DeLano, 2004 ▸). Corresponding color coding is used in (*a*) and (*b*); arbitrary color coding is used in (*c*) and (*d*).

The protein is a *D*_5_-symmetric decamer with close similarity to several orthologous proteins from eubacteria and animals (Fig. 2 ▸, Supplementary Table S1; Nar *et al.*, 1995 ▸). The r.m.s.d. values to previously reported GTP cyclohydrolase I structures were in the range 0.76–1.3 Å for the monomers, whereas r.m.s.d. values in the range 0.12–0.34 Å were found on comparing individual *L. monocytogenes* GTP cyclohydrolase I monomers. However, no appreciable density representing the essential zinc ions was present, and the two canonical cysteine residues Cys78 and Cys150 that form part of the putative zinc-binding site (for a sequence alignment, see Supplementary Fig. S2) were oxidized to the disulfide level; it is most probable that the zinc had been lost owing to the chelating activity of the citrate that was used as a precipitant, and loss of the metal cation had been followed by oxidation of the zinc-chelating cysteine residues. A similar observation had previously been made for crystals of GTP cyclohydrolase I from *E. coli* that had been exposed to EDTA. In comparison, the *Listeria* protein appears to be even more sensitive to chelator-induced zinc-ion loss, but a search for other (nonchelating) crystallization conditions has been unsuccessful to date.

In parallel to the findings for other decameric GTP cyclohydrolases (Auerbach *et al.*, 2000 ▸; Nar *et al.*, 1995 ▸; Tanaka *et al.*, 2005 ▸; Maita *et al.*, 2002 ▸), each of the ten active sites of the protein is located at the interface of three subunits (two in one *C*_5_-symmetric unit and a third in the adjacent *C*_5_-symmetric pentamer unit). The residues lining the active-site cavity of the *L. monocytogenes* protein have high temperature factors, possibly owing to the absence of a divalent cation.

**Figure 3:**
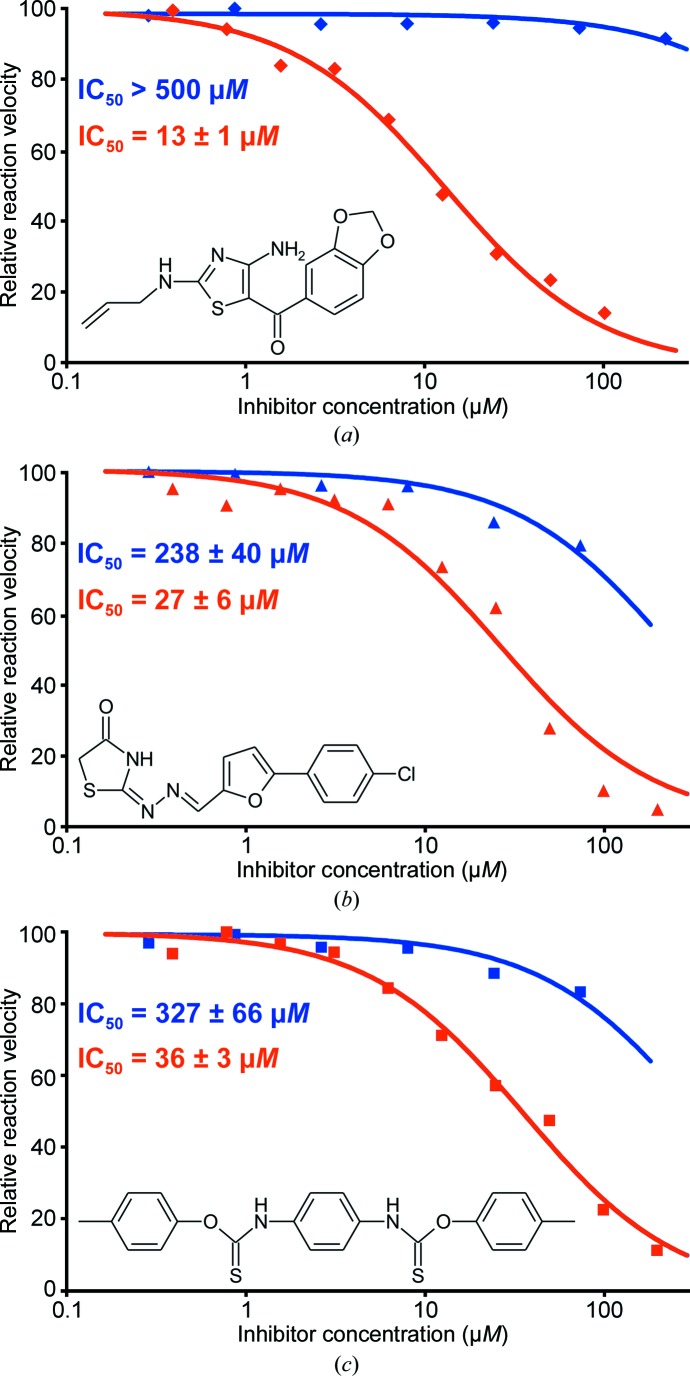
Dose–response curves. Red, GTP cyclohydrolase I from *L. monocytogenes*; blue, human GTP cyclohydrolase I. Inhibition of the *Listeria* enzyme is significantly stronger compared with the human enzyme. IC_50_ values were calculated with *DynaFit* (BioKin, Watertown, Massachusetts, USA; Kuzmič, 1996 ▸).

In order to explore the druggability of the protein, we screened a small library of about 9000 highly diverse compounds (Bracher *et al.*, 1998 ▸). Assays were run in 384-well plates and were monitored photometrically at 330 nm. Hits were rescreened against GTP cyclohydrolase I from *L. monocytogenes* and human GTP cyclohydrolase I, and IC_50_ values were determined using the photometric assay. Semilogarithmic dose–response curves showed symmetrical, S-shaped features, and the overall *Z* factor (Zhang *et al.*, 1999 ▸) of the screen was 0.87. Three heterocyclic compounds inhibited the *L. monocytogenes* enzyme with IC_50_ values in the low double-digit micromolar range while not acting as strong inhibitors of human GTP cyclohydrolase I (Fig. 3 ▸; for details of the high-throughput assay, see the supporting information). Although stronger inhibitors have been reported down to a *K*_i_ of 5.4 n*M* (Tanaka *et al.*, 2005 ▸), all of the inhibitors reported so far are substrate or product analogs.

### 3.2. Discussion   

Animals and many microorganisms feature homodeca­meric, *D*_5_-symmetric GTP cyclohydrolases I with molecular masses of about 200 kDa. The enzyme from *L. monocytogenes* is similar in terms of sequence and three-dimensional structure to orthologs from eubacteria (*E. coli* and *Yersinia pestis*). The structure is also similar to mammalian cyclohydrolase I (rat and mouse), although the mammalian protomers are somewhat longer.

Our data confirm that GTP cyclohydrolase I from *L. monocytogenes* is well suited for high-throughput screening. Importantly, screening does not require any supplementary enzymes, since the enzyme product can be monitored directly by photometry.

Screening a small library of about 9000 structurally diverse compounds retrieved an inhibitor with an IC_50_ of 13 µ*M*; importantly, the compound was much less active against human GTP cyclohydrolase I (IC_50_ > 500 µ*M*).

In order to discuss the potential role of bacterial GTP cyclohydrolase I as an anti-infective drug target, the role of GTP cyclohydrolase I in bacterial pathogens as well as in the human host must be addressed in some detail. Importantly, in 2006 it was found that certain eubacteria do not carry a *folE* gene but produce a homotetrameric protein designated GTP cyclohydrolase IB that is under the control of the *folE2* gene (El Yacoubi *et al.*, 2006 ▸). In contrast to the decameric GTP cyclohydrolases, the type IB protein is not strictly dependent on zinc ions but can use several different divalent transition-metal ions, including iron, for catalysis. In a remarkable parallel to the decameric type I enzymes, the active sites of the type IB enzymes are also located at the interfaces of three adjacent subunits. Moreover, the protein segments that are located at the surface of the active-site cavity align almost perfectly with the cognate sequence elements of GTP cyclohydrolase I, although they have been reshuffled with respect to their location in the overall protein sequence. The *folE2*-type GTP cyclohydrolase provides a metabolic advantage under conditions of limited zinc supply.

The *L. monocytogenes* genome does not contain a *folE2* gene. However, certain other pathogenic eubacteria contain *folE2* genes, either alone or in combination with *folE* and *folE1* genes; multiple genomic copies of *folE* and *folE1* have also been observed. Whether it would be possible to develop inhibitors that act against the type IB as well as the type I GTP cyclohydrolase is as yet unknown. Inhibitors that act only against one type of GTP cyclohydrolase would be significantly restricted with regard to their therapeutic spectrum.

Yet another caveat needs to be considered with regard to the use of GTP cyclohydrolases (type I and/or type IB) as anti-infective drug targets, namely the structural similarity of bacterial GTP cyclohydrolase I to human GTP cyclohydrolase I. The human enzyme catalyzes the first committed step in the biosynthesis of tetrahydrobiopterin that is required for the catabolism of phenylalanine and for the biosynthesis of nitric oxide (for reviews, see Fischer & Bacher, 2005 ▸; Gräwert *et al.*, 2013 ▸). Ideally, any putative drugs directed at microbial GTP cyclohydrolases should be exempt from inhibition of the human ortholog. Nevertheless, the stringency of this argument may not be absolute. In fact, the human enzyme is considered to be a potential target for pain relief (Tegeder *et al.*, 2006 ▸). Although a genetic deficiency of GTP cyclohydrolase causes severe neurological deficits during fetal and childhood development, its inhibition for the duration of treatment of an acute infectious disease in adults for short periods may be acceptable.

Historically, the success of antifolates as anti-infective drugs has benefited massively from the possibility of the simultaneous inhibition of two folate-pathway enzymes, namely dihydro­pteroate synthase and dihydrofolate reductase (Fig. 1 ▸). Despite the caveats addressed above, the possibility of adding a third potential anchor point to the arsenal of antifolate strategies may be worth considering. In this context, it is also relevant to note that fungal protozoan pathogens only have the classical GTP cyclohydrolase I, in contrast to the more complex situation in bacterial pathogens.

## Supplementary Material

PDB reference: GTP cyclohydrolase I, 4uqf

Supporting Methods, Supplementary Figures and Supplementary Table. DOI: 10.1107/S2053230X19010902/no5161sup1.pdf

## Figures and Tables

**Table 1:** Macromolecule-production information Restriction sites are underlined. The first triplet of the *folE* gene and the stop codon are shown in bold. The His_6_ tag, His_6_ tag-coding sequences and enteropeptidase- and enteropeptidase-coding sequences are shown in italics.

Source organism	*L. monocytogenes*
Forward primer	*CACCATCAT*GGTTCCGATGATGACGATAAG**GAG**CAAATAGACAAACAAAAGATTGCTGATGCG
Forward primer II	ATAATAATAGAATTCATTAAAGAGGAGAAATTAACCATG*CATCATCACCACCATCAT*GGTTCCGATGATGACGATAAG
Reverse primer	TATTATTATGGATCC **TTA**ATTATGCTTAATTAAAGCCAAAACTTCACTTCTAAGCTT
Cloning vector	pNCO113
Expression vector	pNCO113
Expression host	*E. coli*
Complete amino-acid sequence of the construct produced	M*HHHHHH*GS*DDDDK*EQIDKQKIADAVKVILEAVGENPDREGLIDTPMRVARMYEEVFAGLKKDPSVHFDTIFEEQHEELVLVKDIRFSSMCEHHLVPFFGVAHVAYLPQNGRVAGLSKLARVVDDVSRRPQLQERITTTVAEIMMEKLKPLGVMVIMEAEHMCMTIRGVNKPGTKTITSAVRGAFKNDDKLRSEVLALIKHN

**Table 2:** Crystallization

Method	Sitting drop
Plate type	96-well
Temperature (K)	283
Protein concentration (mg ml^−1^)	5
Buffer composition of protein solution	10 m*M* Tris–HCl pH 7.0, 75 m*M* NaCl
Composition of reservoir solution	0.1 *M* HEPES pH 7.3, 1.33 *M* sodium citrate
Volume and ratio of drop	2 µl (1:1)
Volume of reservoir (µl)	60

**Table 3:** Data collection and processing

Diffraction source	Beamline P14 (MX2), PETRA III, EMBL c/o DESY
Wavelength (Å)	1.23953
Temperature (K)	100
Detector	Dectris PILATUS 6M
Crystal-to-detector distance (mm)	349.213
Rotation range per image (°)	0.1
Total rotation range (°)	360
Exposure time per image (s)	0.1
Space group	*P*2_1_
*a*, *b*, *c* (Å)	78.25, 141.85, 90.78
α, β, γ (°)	90, 104.61, 90
Mosaicity (°)	0.251
Resolution range (Å)	30.00–2.40 (2.53–2.40)
Total No. of reflections	510889
No. of unique reflections	74531
Completeness (%)	99.7 (99.8)
Multiplicity	6.9 (6.8)
〈*I*/σ(*I*)〉	19.2 (3.5)
*R*_r.i.m._ †	0.076 (0.617)
Overall *B* factor from Wilson plot (Å^2^)	39.9

† Estimated *R*_r.i.m._ = *R*_merge_[*N*/(*N* − 1)]^1/2^, where *N* is the data multiplicity.

**Table 4:** Structure refinement

Resolution range (Å)	87.84–2.40 (2.462–2.400)
Completeness (%)	99.6
No. of reflections, working set	70754 (5214)
No. of reflections, test set	3752 (281)
Final *R*_cryst_	0.193 (0.309)
Final *R*_free_	0.226 (0.314)
Cruickshank DPI	0.25
No. of non-H atoms	
Protein	14475
Water	0
Total	14475
R.m.s. deviations	
Bonds (Å)	0.014
Angles (°)	1.773
Average *B* factors (Å^2^)	
Protein	49.6
Ramachandran plot	
Favored regions (%)	97.7
Additionally allowed (%)	2.1
Outliers (%)	0.2
