# Supplementary material for: Structure of GTP cyclohydrolase I from Listeria monocytogenes, a potential anti-infective drug target
Source: Acta Crystallogr F Struct Biol Commun. 2019 Aug 30;75(Pt 9):586–92. doi: 10.1107/S2053230X19010902 (PMC6718149; doi:10.1107/S2053230X19010902)
Supplement: Supplementary file 1 [file f-75-00586-sup1.pdf]

STRUCTURAL BIOLOGY COMMUNICATIONS

Volume 75 (2019)

Supporting information for article:

Structure of GTP cyclohydrolase I from *Listeria monocytogenes*, a potential anti-infective drug target

Sonja Schüssler, Ilka Haase, Markus Perbandt, Boris Illarionov, Alexandra Siemens, Klaus Richter, Adelbert Bacher, Markus Fischer and Tobias Gräwert

S1. Enzyme Assay

Assay mixtures contained 50 mM Tris hydrochloride, pH 7.5, 100 mM KCl, 200 µM GTP, and protein. They were monitored photometrically at 330 nm.

S2. High throughput screen

A substance library of 9,000 test compounds (courtesy of BASF Crop protection division, Ludwigshafen, Germany) was tested against GTP cyclohydrolase I protein from *L. monocytogenes* in 384 well clear bottom plates (Corning, polystyrol, flat-bottom, non-treated surface). 32 wells of each plate were used as “signal”, i.e. as positive control with enzyme and substrate, but without test compound. 32 wells of each plate were used as “control”, i.e. as negative control with substrate but without enzyme or test compound. Each of the remaining 320 wells contained app. 50 µM test compound (depending on the molecular weight of a given test substance) in 3 µL DMSO. The compounds were pipetted with a Hamilton Starlet robot (Hamilton Germany GmbH, Planegg, Germany) with a 96-tip pipetting block. Enzyme (final concentration 8.7 µM) and substrate (final concentration 200 µM) were supplied in separate solutions that both contained 50 mM Tris hydrochloride, pH 7.5 and 100 mM KCl. 30 µL per well of both solutions were filled into the assay plates with an 8-fold dispenser (Bio Tek Germany, Bad Friedrichshall, Germany) and the plates were then centrifuged for 1 min at 1,000 rpm and room temperature. The reaction was monitored with a SpectraMax M5 plate reader photometer (Molecular Devices GmbH, Biberach a. d. Riss, Germany) at 330 nm and room temperature for 30 min.

Test compounds of a respective plate that lowered the reaction velocity to 70% or less as compared to the signal of the same plate were subjected to IC_{50} determination. Therefore, 8 concentrations of each test compound at a range of 200 µM to 90 nM were assayed in 96 well plates (Nunc, polystyrol, flat-bottom, non-treated surface) with a final volume of 200 µL per well under the above mentioned conditions. IC_{50} values were calculated using Dynafit (Biokin Ltd., Watertown, Massachusetts, USA). Counterscreens against human GTP cyclohydrolase I protein were conducted under the same conditions as the initial screen but with an enzyme concentration of 5.9 µM.

S3. Analytical ultracentrifugation

Hydrodynamic studies were performed with an analytical ultracentrifuge Optima XL-A (Beckman Coulter GmbH, Krefeld, Germany) equipped with absorbance optics. Samples contained 50 mM Tris hydrochloride, pH 7.5, 250 mM NaCl, 2 mM dithiothreitol, and protein.

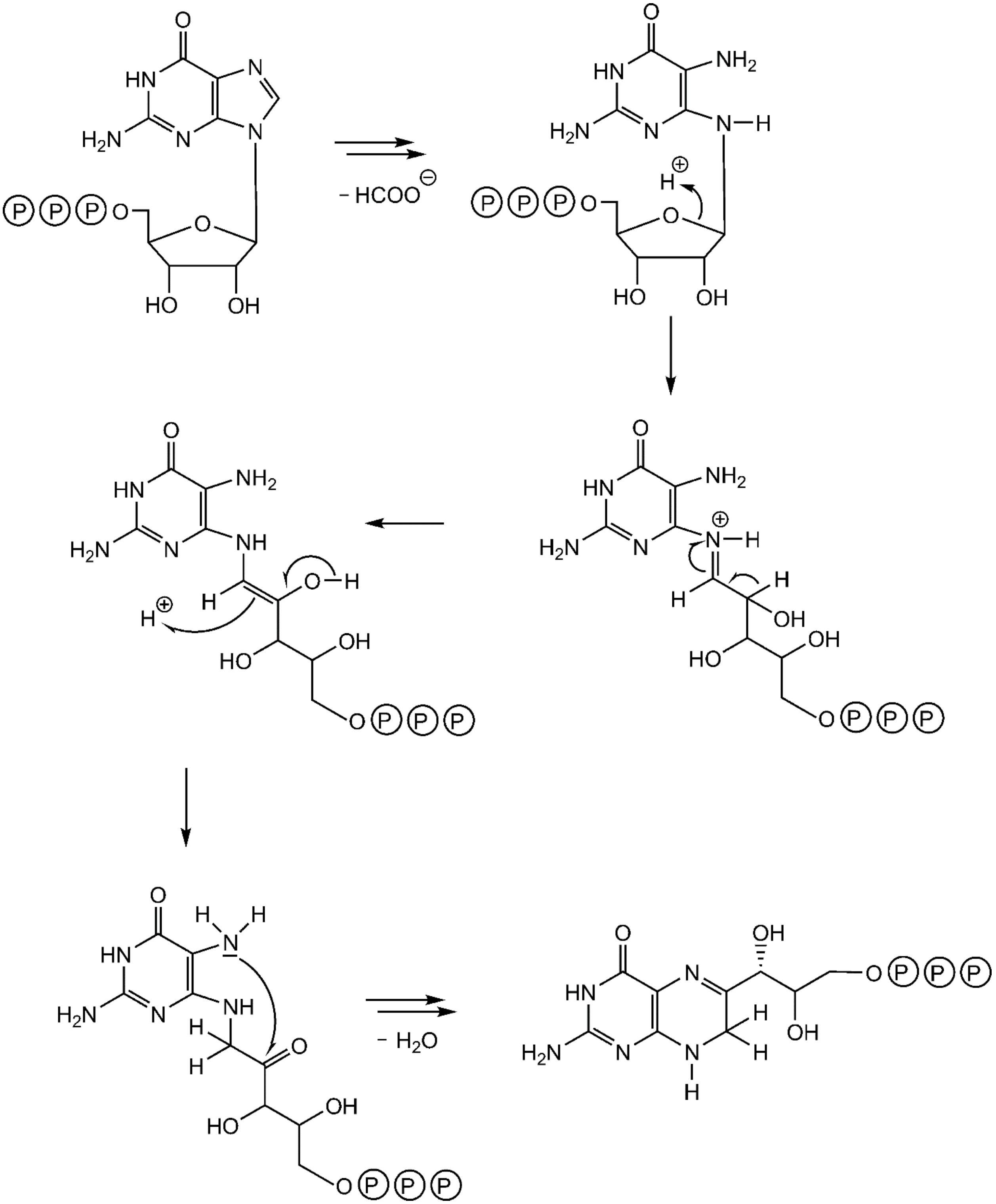

Figure S1 Putative reaction trajectory of GTP cyclohydrolase I.

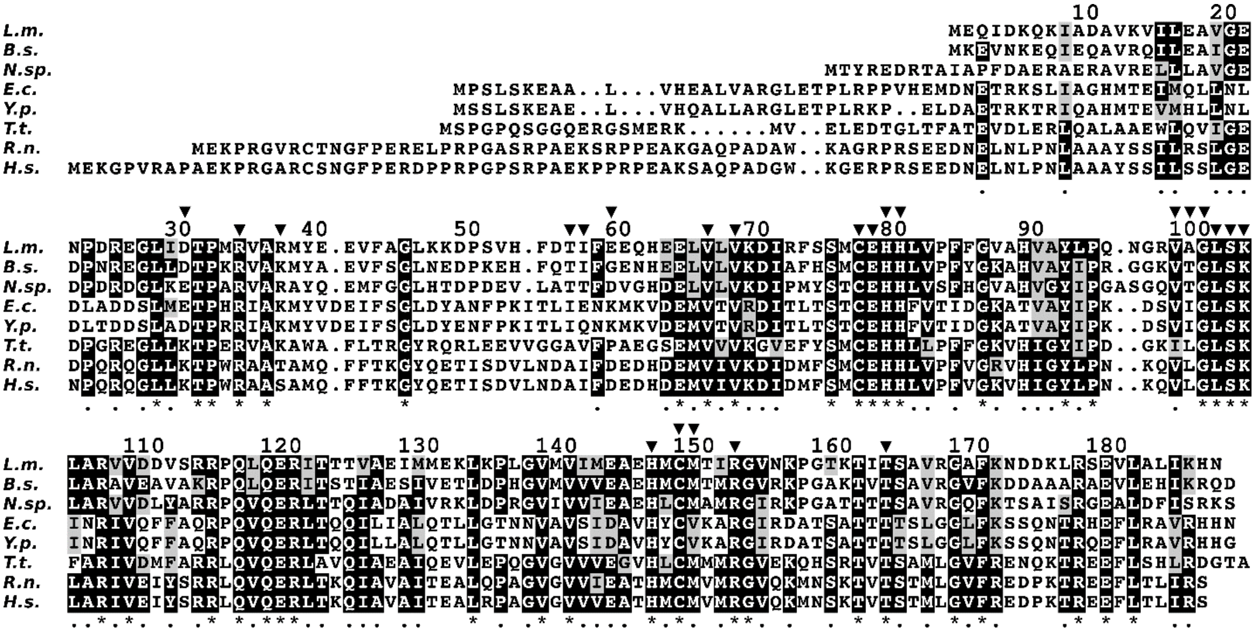

Figure S2 Sequence alignment of GTP-CHI proteins from various species. Sequence numbering according to *L. monocytogenes*. Triangles: active-site amino acids. Asterisks: stringently conserved amino acids. *L.m.*: *Listeria monocytogenes*. *B.s.*: *Bacillus subtilis. N.sp.*: *Nocardia* sp.. *E.c.*: *Escherichia coli*. *Y.p.*: *Yersinia pestis*. *T.t.*: *Thermus thermophilus*. *R.n.*: *Rattus norvegicus*. *H.s.*: *Homo sapiens*.

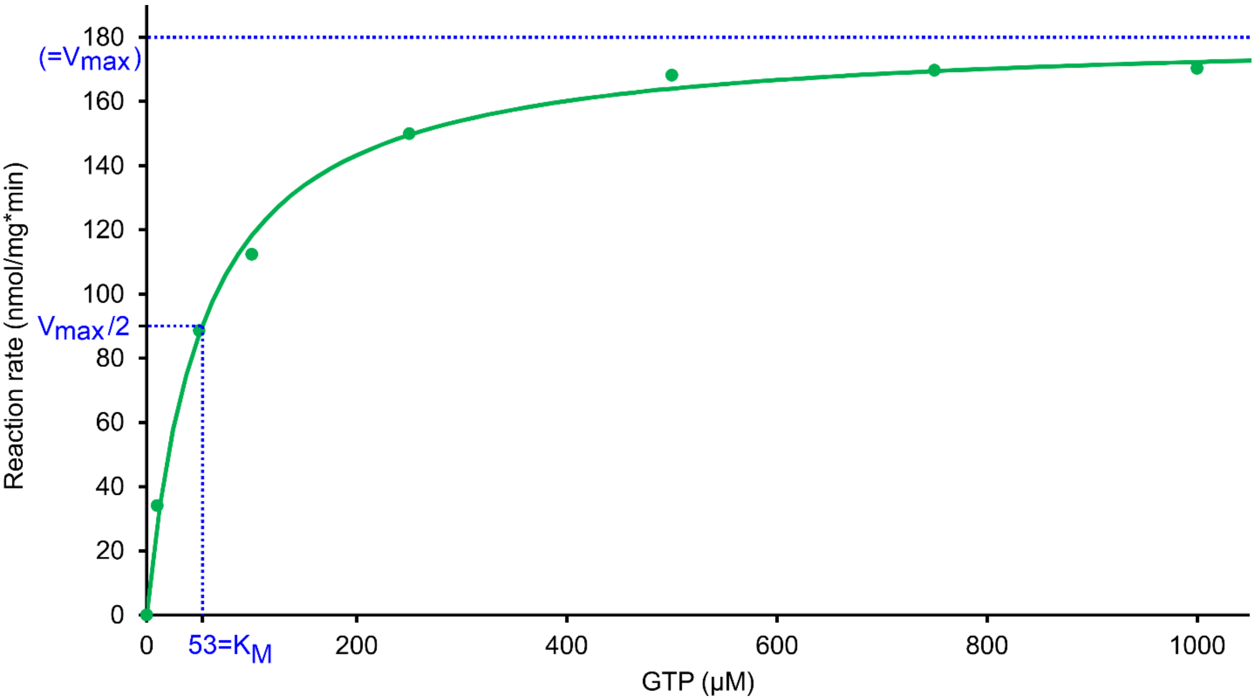

Figure S3 Michaelis-Menten kinetics of GTP cyclohydrolase I from Listeria monocytogenes. Kinetic properties were determined with *DynaFit*.

Table S1 *Listeria monocytogenes* versus other eubacteria and animals

RMSD values for various structural superpositions in Å.

RCSB entry code	Organism	RMSD Monomer	…using x atoms	RMSD Monomer cα	...using x atoms	RMSD Decamer	…using x residues
1GTP	*E. coli*	1.25	985	1.21	158	3.65	344
1FBX		1.23	976	1.16	154	4.04	200
1A8R		1.13	955	1.06	152	1.78	184
1A9C		1.20	967	1.14	154	1.83	184
1N3T		1.22	974	1.19	158	5.94	208
1N3R		1.22	978	1.18	158	2.18	368
1N3S		0.99	907	0.83	140	6.81	200
4DU6	*Y. pestis*	1.18	929	1.15	152	1.95	368
1WPL	*R. norvegicus*	0.85	995	0.80	160	1.39	368
1IS7		0.76	969	0.70	157	1.33	368
1IS8		0.76	978	0.72	159	1.50	360
1WM9	*T. thermophilus*	1.04	1007	0.95	155	2.95	376
1FB1	*H. sapiens*	0.78	966	0.68	156	1.24	368

Superpositions were done with Pymol using the commands “align” for monomers and “cealign” for decamers.
